# *MTHFR* Gene Polymorphism Association With Psoriatic Arthritis Risk and the Efficacy and Hepatotoxicity of Methotrexate in Psoriasis

**DOI:** 10.3389/fmed.2022.869912

**Published:** 2022-04-11

**Authors:** Jie Zhu, Zhicheng Wang, Lu Tao, Ling Han, Qiong Huang, Xu Fang, Ke Yang, Guiqin Huang, Zhizhong Zheng, Nikhil Yawalkar, Zhenghua Zhang, Kexiang Yan

**Affiliations:** ^1^Shanghai Institute of Dermatology, Department of Dermatology, Huashan Hospital, Fudan University, Shanghai, China; ^2^Department of Laboratory Medicine, Huashan Hospital, Fudan University, Shanghai, China; ^3^Department of Information, Huashan Hospital, Fudan University, Shanghai, China; ^4^Department of Dermatology, Inselspital, Bern University Hospital, University of Bern, Bern, Switzerland

**Keywords:** methylenetetrahydrofolate reductase, psoriasis, psoriatic arthritis, methotrexate, biomarker, pharmacogenetics

## Abstract

**Aims:**

To assess whether *MTHFR* rs1801131 and rs1801133 SNPs are associated with concomitant psoriatic arthritis (PsA) and investigate the efficacy and hepatotoxicity of MTX in patients with psoriasis in the Han Chinese population.

**Methods:**

This prospective, single-arm, interventional study recruited a total of 309 patients with psoriasis, 163 with psoriatic arthritis and 146 without psoriatic arthritis, who completed a 12-week MTX treatment and 1,031 healthy controls. Patients' characteristics including age, gender, disease duration, height, weight, smoking status, alcohol consumption, medical history, disease severity and liver function test results were accessed and recorded. Single nucleotide polymorphism (SNP) genotyping of rs1801131 and rs1801133 in the *MTHFR* gene was performed.

**Results:**

The rs1801133 CC genotype was more frequent in patients with PsA than those with PsO and healthy controls (42.3% vs. 28.8% vs. 33.1%, *p* < 0.05). The 90% reduction from baseline PASI score (PASI 90) response rates to MTX were significantly higher in patients with the rs1801133 TT genotype than those with the CT and CC genotype (33.96% vs. 19.31% vs. 14.41%, *OR* = 2.76, *p* = 0.006). The rs1801133 CT+TT genotype was more frequent in PsA patients with abnormal liver function than in those with normal liver function (*p* < 0.05). In addition, patients with the rs1801131 CT genotype had lower PASI 75 response rates to MTX (*OR* = 0.49, *p* = 0.01), and lower risk of ALT elevation (*OR* = 0.46, *p* = 0.04).

**Conclusions:**

This study provided some evidence for *MTHFR* polymorphism association with the risk of PsA and the efficacy and hepatotoxicity of the low-dose MTX in the Chinese population. Given the relatively small sample size and potentially missed diagnosis of PsA, the results from this study warrant further investigation.

## Introduction

Psoriasis is a chronic, immune-mediated, multisystemic, inflammatory disease caused by the interaction of multiple susceptibility genes and environmental factors that affects 1–3% of the population worldwide (1). Psoriatic arthritis (PsA) most commonly develops in 20–30% of psoriatic patients with preceding psoriasis or develops concomitantly with psoriasis, and it manifests all features of arthritis, including enthesitis (2). Our previous study has demonstrated that methotrexate (MTX), an efficacious treatment for both psoriasis and PsA but associated with hepatotoxicity, is more effective and safer in patients with psoriasis without arthritis (PsO) than in PsA patients (3). Therefore, it is critical to identify biomarkers for predicting the development of PsA and the efficacy and hepatotoxicity of MTX.

Methylenetetrahydrofolate reductase (MTHFR) is a crucial enzyme in homocysteine/methionine metabolism that catalyzes the formation of 5-methyltetrahydrofolate, which is the methyl donor for the synthesis of methionine from homocysteine (Hcy) (4). The 677 C>T mutation (rs1801133) in MTHFR disrupts its thermostability, leading to defective enzyme activity and elevation of Hcy levels (5). Individuals who are homozygous for the 677T allele have only 32% of the MTHFR enzymatic activity measured in individuals homozygous for the wild-type C allele, whereas heterozygotes retain 64% of the wild-type MTHFR enzymatic activity. In contrast, homozygosity in the 1298 A>C mutation (rs1801131) does not lead to higher Hcy levels (6).

The association between *MTHFR* polymorphism and the susceptibility to psoriasis has been supported in Caucasians, Turkish, and Chinese populations (7–12). A study carried out in China reported that the rs1801133 T allele and the rs1801131 C allele were all associated with increased psoriasis risk (9). However, no studies have been found that surveyed the frequency of *MTHFR* polymorphisms in patients with PsA in the Han Chinese populations. In addition, only two studies have attempted to investigate the correlation between *MTHFR* polymorphisms and MTX efficacy in psoriasis, both suggesting that no significant genotypic associations were found (13, 14). As for the hepatotoxicity of MTX, according to Campalani et al. (14), patients with the rs1801131 C allele were less likely to develop hepatotoxicity, and no association was detected between the rs1801133 and hepatotoxicity of MTX.

In the present study, we sought to evaluate whether *MTHFR* rs1801131 and rs1801133 SNPs are associated with concomitant PsA, the efficacy, and hepatotoxicity of MTX in patients with psoriasis in the Han Chinese population.

## Materials and Methods

### Subjects

This single-center, prospective trial was conducted at the Department of Dermatology, Huashan Hospital, Fudan University in Shanghai, China, between 1 April 2015 and 15 October 2018. Patients 18 years or older were recruited from the outpatient population. Patients who received therapy with UV light, methotrexate, or other systemic treatments for psoriasis within 1 month of study initiation were excluded. Topical treatments were stopped 1 week before the start of the methotrexate treatment. The study was conducted in accordance with the Declaration of Helsinki. The Medical Ethics Committee of Huashan Hospital, Fudan University, reviewed and approved the protocol (approval number: MTX201501), and all participants provided written informed consent. The diagnosis of PsO and PsA was based on typical clinical and/or histopathological criteria and the ClASsification criteria for Psoriatic ARthritis (CASPAR), respectively. Enthesitis and joint involvement were usually detected using magnetic resonance imaging (MRI) and ultrasonography in our clinic. No radiographs were available, and therefore evidence of juxta-articular new bone formation was excluded from the assessment of CASPAR in the present study. All patients and healthy controls were unrelated Chinese Han individuals. All patients were interviewed, and data regarding the age at disease onset, smoking status, alcohol consumption, and medical history (including hypertension and diabetes mellitus) were recorded. Two certified dermatologists graded psoriasis using the Psoriasis Area and Severity Index (PASI) and body surface area (BSA) scores at the baseline and at weeks 4, 8, and 12. Genomic DNA was extracted from whole blood samples collected from each patient and healthy control.

### Treatment Strategy and Hepatotoxicity Evaluation

Patients were treated with low-dose MTX monotherapy. The initial MTX treatment comprised a weekly oral dose of 7.5–10 mg. The dose was increased by 2.5 mg every 2–4 weeks to a maximum of 15 mg weekly based on the patients' clinical response, adverse events, and laboratory assessment results. If liver enzyme (AST/ALT) elevations were greater than two-fold and less than three-fold of the upper normal limit, the MTX dose was reduced by 2.5 mg weekly and administered again between 2 and 4 weeks. If liver enzyme elevations were greater than three-fold of the upper normal limit, MTX treatment was stopped. Liver function tests in the present study included measurement of ALT, AST, direct bilirubin (DBIL), and total bilirubin (TBIL) levels. Abnormal liver function was considered if there was an elevation above the normal upper limit of any one of the above four enzyme levels.

### DNA Extraction and Genotyping Analysis

EDTA-anticoagulated whole blood (5 mL) samples were collected from all patients and stored at −80°C. Genomic DNA was extracted from peripheral blood lymphocytes using the FlexiGene DNA Purification Kit (Qiagen, Hiden, Germany) according to the manufacturer's instructions and diluted to 20 ng/μL. The rs1801133 and rs1801131 single nucleotide polymorphism (SNP) of *MTHFR* was genotyped using SequenomMassARRAY with the SequenomMassARRAY Assay Design 3.0 software used to design the polymerase chain reaction (PCR) and detection primers. The PCR products were subsequently used as templates for locus-specific single-base extension reactions. The resulting products were desalted and transferred to a 384-element SpectroCHIP array (Sequenom Inc.). Matrix-assisted laser desorption/ionization-time of flight mass spectrometry (Sequenom Inc.) was used for allele detection. The mass spectrograms were analyzed using the MassARRAY Type software (Sequenom Inc.). We performed quality control of SNPs and samples at a call rate of 99.7%. The genotype distributions and allele frequencies for *MTHFR* gene SNP rs1801131 and rs1801133 were shown in Supplementary Table 2. The genotype frequencies of all subjects comply with Hardy-Weinberg equilibrium (*p* > 0.05).

### Statistical Analysis

Continuous variables were summarized as mean ± SD (standard deviation), and categorical variables as numbers and percentages. One-way ANOVA analysis (Kruskal-Wallis test or Newman-Keuls Comparison Test), Pearson χ2 test, and Fisher's exact test were used when appropriate. The effect size was calculated using an online Cramer's V calculator (https://mathcracker.com/cramers-v-calculator?). To explore the factors independently associated with concomitant PsA and efficacy and hepatotoxicity of MTX, we conducted a stepwise multivariate regression analysis using SPSS ver. 23.0 software (SPSS Inc., Chicago, IL, USA) and presented the final model. A value of *p* < 0.05 was considered to indicate statistical significance.

## Results

### Clinical Characteristics and Main Outcomes According to rs1801133 and rs1801131 Genotype

Out of the 325 patients who were treated with MTX monotherapy, 310 patients completed the 12-week MTX treatment and at least 1-year duration of monthly follow-up. Six and nine patients discontinued treatment at weeks 4 and 8, respectively. None of the patients withdrew owing to adverse effects. Three hundred and nine of the 310 patients were determined the single nucleotide polymorphism genotype of rs1801133 and rs1801131 in *MTHFR* and 1031 of the 1031 healthy controls, respectively. A total of 163 of the 309 patients fulfilled the CASPAR criteria and were classified with PsA. A complete description of the clinical characteristics of 163 PsA patients is described in Supplementary Table 1.

Table 1 shows the clinical characteristics of the 309 patients with psoriasis according to rs1801133 genotype and rs1801131 genotype. Patients with the rs1801133 CC genotype had a higher incidence of concomitant PsA (*p* = 0.036) and were older (*p* = 0.049) than those with the CT and TT genotype. In contrast, patients with the rs1801133 TT genotype had higher PASI 90 response rates to MTX (*p* = 0.013) and higher PASI score at baseline (*p* = 0.045) than those with the CT and CC genotype. Considering rs1801131, CT genotype carriers had lower PASI 75 response rates to MTX (*p* = 0.025) and lower incidence of ALT liver elevation (*p* = 0.033) than those with the CC and TT genotype. In addition, the corresponding effect sizes for PASI 75 and PASI 90 response rates were 0.154 and 0.167, respectively.

**Table 1:** Clinical characteristics of 309 patients with psoriasis according to rs1801133 genotype and rs1801131 genotype.

		**rs1801133**				**rs1801131**		
**Variables**	**TT (*n* = 53)**	**CT (*n* = 145)**	**CC (*n* = 111)**	***p*-value*^a^***	**TT (*n* = 213)**	**CT (*n* = 88)**	**CC (*n* = 8)**	***p*-value*^a^***
Male, n (%)	38 (71.70)	99 (68.28)	80 (72.07)	0.779	148 (69.48)	63 (71.59)	6 (75.00)	0.895
Age, years, mean ± SD	47.42 ± 14.98	46.60 ± 15.80	50.98 ± 13.66	**0.049**	48.37 ± 15.28	47.32 ± 14.59	54.13 ± 12.86	0.457
Age at disease onset, years, mean ± SD	33.08 ± 15.14	33.48 ± 15.76	37.48 ± 15.63	0.086	34.85 ± 16.18	34.33 ± 14.31	40.38 ± 17.75	0.582
BMI (kg/m^2^), mean ± SD	24.48 ± 3.75	24.19 ± 3.42	24.56 ± 3.42	0.694	24.37 ± 3.48	24.53 ± 3.59	23.97 ± 2.27	0.859
Nail involvement at baseline, n (%)	41 (77.4)	101 (69.7)	66 (59.5)	0.052	146 (68.5)	58 (65.9)	4 (50.0)	0.518
Nail involvement at week 12, n (%)	41 (77.4)	100 (69.0)	75 (67.6)	0.417	149 (70.0)	61 (69.3)	6 (75.0)	0.945
PASI at baseline, mean ± SD	15.83 ± 6.83	13.82 ± 7.63	13.62 ± 7.60	**0.045**	14.25 ± 7.58	13.72 ± 7.29	13.98 ± 8.85	0.857
BSA at baseline, mean ± SD	31.77 ± 18.56	29.34 ± 22.68	26.76 ± 19.55	0.188	29.30 ± 21.26	27.74 ± 19.57	25.08 ± 14.14	0.738
MTX cumulative dosage, mg, mean ± SD	133.3 ± 18.00	136.2 ± 23.18	135.5 ± 19.97	0.700	135.21 ± 22.06	135.57 ± 19.41	140 ± 18.32	0.821
PASI 50 achievements at week 12, n (%)	41 (77.36)	103 (71.03)	75 (67.57)	0.434	160 (75.12)	54 (61.36)	5 (62.50)	0.050
PASI 75 achievements at week 12, n (%)	31 (58.49)	66 (45.52)	46 (41.44)	0.119	109 (51.17)	30 (34.09)	4 (50.00)	**0.025**
PASI 90 achievements at week 12, n (%)	18 (33.96)	28 (19.31)	16 (14.41)	**0.013**	48 (22.54)	13 (14.77)	1 (12.50)	0.268
Hypertension, n (%)	17(32.08)	47 (32.41)	49 (44.14)	0.117	69 (32.39)	40 (45.45)	4 (50.00)	0.074
Diabetes, n (%)	11(20.75)	26 (17.93)	21 (18.91)	0.902	42 (19.72)	15 (17.05)	1 (12.50)	0.777
Smoking, n (%)	22 (41.51)	41 (28.28)	38 (34.23)	0.194	71 (33.33)	25 (28.41)	5 (62.50)	0.135
Alcohol consumption, n (%)	11 (20.75)	32 (22.07)	29 (26.13)	0.667	52 (24.41)	17 (19.32)	3 (37.50)	0.400
Arthritis, n (%)	23 (43.40)	71 (48.97)	69 (62.16)	**0.036**	113 (53.05)	45 (51.14)	5 (62.50)	0.817
ALT level elevation, n (%)	14(26.42)	34 (23.45)	19 (17.11)	0.312	53 (24.88)	11 (12.5)	3 (37.50)	**0.033**
Abnormal liver function, n (%)	15 (28.30)	40 (27.59)	24 (21.62)	0.490	61 (28.64)	15 (17.05)	3 (37.50)	0.082

*ALT, alanine transaminase; BMI, body mass index (calculated as weight in kilograms divided by height in meters squared); PASI, Psoriasis Area Severity Index; BSA, body surface area; MTX, methotrexate; PASI 50, 50% reduction from baseline PASI score; PASI 75, 75% reduction from baseline PASI score; PASI 90, 90% reduction from baseline PASI score.*

a *One-way ANOVA analysis (Kruskal-Wallis test or Newman-Keuls Comparison Test), Pearson χ^2^ test, Fisher's exact test were used when appropriate. Bold represents significant difference (p < 0.05)*.

Associations between the *MTHFR* rs1801133 genotype and PsA were further shown in Table 2. The frequency of C allele in PsA was higher than that in PsO and healthy controls (64.1% vs. 54.1%, 64.1% vs. 56.9%, *p* < 0.05, respectively). Correspondingly, the rs1801133 CC (wild) genotype was more frequent in patients with PsA than PsO and healthy controls (42.3% vs. 28.8%, *p* < 0.05; 42.3% vs 33.1%, *p* < 0.05, respectively). In contrast, no different rs1801133 genotype distribution was observed between PsO and healthy controls.

**Table 2:** The distribution of *MTHFR* SNP rs1801133 in patients with PsO and patients with PsA and the healthy controls.

	**No. (%) of Patients**					
				**PsA vs. HC**	**PsO vs. HC**	**PsA vs. PsO**
	**PsA (*n* = 163)**	**PsO (*n* = 146)**	**HC (*n* = 1031)**	***P*-value*^a^***	***P*-value*^a^***	***P*-value*^a^***
**Allele**
C allele	209 (64.1)	158 (54.1)	1174 (56.9)			
T allele	117 (35.9)	134 (45.9)	888 (43.1)	**0.0157**	0.3774	**0.0138**
**Genotype**
CC	69 (42.3)	42 (28.8)	341 (33.1)			
CT	71 (43.6)	74 (50.7)	492 (47.7)			
TT	23 (14.1)	30 (20.5)	198 (19.2)	**0.0497**	0.5821	**0.0362**
**Dominant Model**
CC	69 (42.3)	42 (28.8)	341 (33.1)			
CT + TT	94 (57.7)	104 (71.2)	690 (66.9)	**0.0262**	0.3453	**0.0174**
**Recessive Model**
CC + CT	140 (85.9)	116 (79.5)	833 (80.8)			
TT	23 (14.1)	30 (20.5)	198 (19.2)	0.1293	0.7371	0.1734

*PsA, patients with psoriatic arthritis; PsO, patients with psoriasis without psoriatic arthritis; HC, healthy controls.*

a *P values determined with the χ 2 test or Fisher exact test, as appropriate. Bold represents significant difference (p < 0.05)*.

### Stepwise Multivariate Logistic Regression Analysis of Factors Associated With Concomitant PsA and the Efficacy and Hepatotoxicity of MTX

To further investigate the factors independently related to concomitant PsA and efficacy and hepatotoxicity of MTX, stepwise multivariate logistic regression analysis was used, and final models are shown in Table 3. Older age was independently associated with higher incidence of concomitant PsA (OR 1.027, 95% CI 1.011–1.044, *p* = 0.001), as was the rs1801133 CC genotype (OR 1.651, 95% CI 1.017–2.680, *p* = 0.043). Higher BMI, lower PASI at baseline, and concomitant PsA were significantly associated with lower PASI 75 and PASI 90 response rates to MTX (*p* < 0.05). In addition, the OR for patients with the rs1801131 CT genotype to achieve PASI 75 was 0.492 (95% CI = 0.280–0.865, *p* = 0.014), while the OR for those with rs1801133 TT genotype to achieve PASI 90 was 2.759 (95% CI = 1.336–5.699, *p* = 0.006). Younger age, higher BMI, and smoking increased the risk of MTX-associated ALT elevation and abnormal liver function (*p* < 0.05). Moreover, BSA at baseline was positively associated with ALT elevation (OR = 1.015), and the rs1801131 CT genotype was negatively associated with ALT elevation (OR = 0.456).

**Table 3:** Stepwise multivariate logistic regression analysis of factors associated with concomitant PsA, the efficacy and hepatotoxicity of MTX.

	**Predictors**	**OR**	**95% CI**	***p*-value**
Arthritis	Age	1.027	(1.011–1.044)	0.001
	rs1801133 CC	1.651	(1.017–2.680)	0.043
PASI 75 response rates	Age	1.031	(1.013–1.049)	0.001
	PASI at baseline	1.047	(1.014–1.082)	0.006
	BMI	0.888	(0.823–0.958)	0.002
	Arthritis	0.526	(0.317–0.872)	0.013
	rs1801131 CT	0.492	(0.280–0.865)	0.014
PASI 90 response rates	Age at onset	1.030	(1.010–1.050)	0.003
	PASI at baseline	1.066	(1.024–1.109)	0.002
	BMI	0.873	(0.755–0.929)	0.001
	Arthritis	0.361	(0.186–0.700)	0.003
	rs1801133 TT	2.759	(1.336–5.699)	0.006
ALT elevation	Age	0.975	(0.954–0.997)	0.023
	BSA at baseline	1.015	(1.001–1.029)	0.032
	BMI	1.147	(1.053–1.250)	0.002
	Smoking	3.608	(1.960–6.642)	<0.001
	rs1801131 CT	0.456	(0.215–0.964)	0.040
Abnormal liver function*^a^*	Age	0.980	(0.961–0.999)	0.041
	BMI	1.110	(1.028–1.199)	0.008
	Smoking	2.533	(1.455–4.408)	0.001

*ALT, alanine transaminase; BMI, body mass index; BSA, body surface; PASI, Psoriasis Area Severity Index; CI, confidence interval; PASI 75, 75% reduction from baseline PASI score; PASI 90, 90% reduction from baseline PASI score.*

a *Abnormal hepatic function included methotrexate-induced elevation of aspartate transaminase, aspartate transaminase, direct bilirubin, and total bilirubin*.

### The *MTHFR* rs1801133 CT and TT Genotype Was Associated With a Higher Risk of MTX-Induced Abnormal Liver Function in Patients With PsA, Not in Patients With PsO

After stratification for PsO and PsA, as shown in Table 4, the frequency of the rs1801133 T allele was found higher in PsA patients with abnormal liver function than in those with normal liver function (68.9% vs. 53.1%, *p* = 0.0081). Correspondingly, the rs1801133 CT + TT genotype was more frequent in PsA patients with abnormal liver function than in those with normal liver function (49.1% vs. 26.5%, *p* < 0.05). In contrast, no associations were observed between the rs1801133 genotype and abnormal liver function in patients with PsO.

**Table 4:** Association of *MTHFR* rs1801133 polymorphisms with MTX-induced abnormal hepatic function in PsA patients and PsO patients.

	**Patients With PsA (*n* = 163)**				**Patients With PsO (*n* = 146)**			
	**Abnormal hepatic function*^b^*, n (%)**	**Normal hepatic function, n (%)**	**Odds ratio** **(95% CI)**	***P*-value*^a^***	**Abnormal hepatic function*^b^*, n (%)**	**Normal hepatic function, n (%)**	**Odds ratio** **(95% CI)**	***P*-value*^a^***
**Allele**
C	52 (53.1)	157 (68.9)	Reference: 1		33 (61.1)	125 (52.5)	Reference: 1	
T	46 (46.9)	71 (31.1)	1.96 (1.19–3.21)	**0.0081**	21 (38.9)	113 (47.5)	0.70 (0.38–1.28)	0.2909
**Genotype**
CC	13 (26.5)	56 (49.1)	Reference: 1		11 (40.7)	31 (26.1)	Reference: 1	
CT	26 (53.1)	45 (39.5)	2.49 (1.19–5.35)		11 (40.7)	63 (52.9)	0.49 (0.19–1.28)	
TT	10 (20.4)	13 (11.4)	3.31 (1.25–8.66)	**0.0229**	5 (18.5)	25 (21.0)	0.56 (0.19–1.81)	0.3067
**Dominant Model**
CC	13 (26.5)	56 (49.1)	Reference: 1		11 (40.7)	31 (26.1)	Reference: 1	
CT + TT	36 (73.5)	58 (50.9)	2.67 (1.31–5.37)	**0.0093**	16 (59.2)	88 (73.9)	0.51 (0.21–1.20)	0.1582
**Recessive Model**
CC + CT	39 (79.6)	101 (88.6)	Reference: 1		22 (81.4)	94 (79.0)	Reference: 1	
TT	10 (20.4)	13 (11.4)	1.99 (0.78–4.63)	0.1453	5 (18.5)	25 (21.0)	0.85 (0.33–2.31)	>.9999

*PsA, patients with psoriatic arthritis; PsO, patients with psoriasis without psoriatic arthritis.*

a *P values determined with the χ 2 test or Fisher exact test, as appropriate.*

b *Abnormal hepatic function included methotrexate-induced elevation of aspartate transaminase, aspartate transaminase, direct bilirubin, and total bilirubin. Bold represents significant difference (p < 0.05)*.

## Discussion

Previous studies have revealed dozens of genes with genomic variations associated with PsA, such as *HLA-C, HLA-B, IL12B, IL23R*, and *REL* (15). To our knowledge, this is the first study to assess the potential association between *MTHFR* polymorphism and the susceptibility to PsA in the Han Chinese population. In the present study, the frequency of the rs1801133C allele in PsA was 64.1%, and the frequency of rs1801133 CC genotype in PsA was 42.3%, which was significantly higher than that in PsO (28.8%) and healthy controls (33.1%). In contrast to the studies on patients with psoriasis (11, 12), we found no difference in the rs1801133 genotype distribution between PsO and healthy controls; this inconsistency may be due to those studies combined PsA and PsO as a whole group when analyzing. It is reported that the association between the SNP rs1801133 and bone phenotypes depends on folate status (16). Since PsA is characterized by both extensive resorption and exuberant new bone formation (17), one possible mechanism by which rs1801133 affect the development of PsA is that SNP rs1801133 modulates serum homocysteine concentrations and leads to subsequent effects on bone. In addition, patients with rheumatoid arthritis (RA) were reported to have a higher frequency of the rs1801133 T allele (18, 19), which is contrary to our observation that the C allele was more frequent among patients with PsA. This finding further provides evidence for different genetics of RA and PsA.

Previous studies reported no associations between SNPs in *MTHFR* and MTX efficacy in psoriasis (13, 14). In the present study, we found psoriatic patients with the rs1801133 TT genotype had higher PASI 90 response rates to MTX, and patients with the rs1801131 CT had lower PASI 75 response rates to MTX. Furthermore, stepwise multivariate logistic regression analysis confirmed that the odds ratio for patients with the rs1801133 TT genotype to achieve PASI 90 was 2.759, and the odds ratio for those with the rs1801131 CT genotype to achieve PASI 75 was 0.492. One possible explanation might be that the reduction of the MTHFR enzyme activity and subsequently higher Hcy level caused by the 677C>T mutation (rs1801133), not the 1298 A>C mutation (rs1801131), increases MTX sensitivity via the inhibition of S-adenosylmethionine and *de novo* purine synthesis (20, 21). The decreased DNA methylation exhibited by the rs1801133 TT genotype also increases the anti-proliferative and anti-inflammatory effects of MTX (22). Nevertheless, it should be noted that the efficacy of MTX was reported to be associated with the dose and the length of therapy (23), and therefore all interpretations regarding high-dose MTX must be made with great caution. Also, the effect sizes for PASI 75 and PASI 90 were relatively small, suggesting that the associations between SNPs in *MTHFR* and MTX efficacy may be weak.

Apart from SNPs in *MTHFR*, stepwise multivariate logistic regression analysis also showed other independent factors associated with the efficacy and hepatotoxicity of MTX in 309 psoriatic patients. Lower PASI 75 and PASI 90 response rates to MTX were both significantly associated with higher BMI and concomitant PsA; this was in accordance with our previous report showing that MTX was more effective in patients with PsA than in those with PsO (3). In addition, smoking and high BMI are crucial factors increasing MTX-related hepatotoxicity in the present study, which is also in line with our earlier observations (3).

In the present study, in PsA, patients with the rs1801133 CT and TT genotype had a higher risk of abnormal liver function than those with the CC genotype. A similar finding also reported that rs1801133 TT genotype might be associated with MTX-induced hepatotoxicity in PsA (24). However, no associations between rs1801133 genotype and abnormal liver function in PsO were found in the present study. This result may be explained by the different immune responses among patients with PsA and PsO. For example, both TNF and IL-17 inhibitors can significantly relieve joint pain, but IL-17 inhibitors lead to better improvement of psoriatic skin lesions, suggesting the different immune-pathogenesis between PsA and PsO. Hepatic ischemia-reperfusion stimulates Kupffer cells and initiates injury through TNF-α upregulation (25), and anti-TNF-α agents have been reported to increase liver enzyme levels (26). This evidence suggests TNF-α in PsA might be involved in liver injury.

Our study has several limitations. The major limitation is the relatively small sample size and the single-center setting. Because of the nature of our clinic, which is specialized in PsA, the proportion of patients with PsA in the cohort was higher than the reported incidence of PsA (20–30% of patients with psoriasis), and the disease tended to be more severe. Additionally, evidence of juxta-articular new-bone formation was excluded from the assessment of CASPAR and the follow-up was relatively short [psoriasis precedes arthritis by an average of 7 years (27)], leading to a potentially missed diagnosis of PsA. Also, given the small number of TT genotype carriers of rs1801131, results regarding rs1801131 should be interpreted with caution. It is clear that further validation of these preliminary findings is necessary. As a first step, the reproducibility of the results should be substantiated in larger multi-center studies. Then, it would be appropriate to investigate whether this genetic marker could been combined with other predictors. Furthermore, digital medicine such as machine learning algorithms might be applied to these data to provide a molecular fingerprint composed of the differential regulation of multiple markers to predict the risk of PsA and the efficacy and hepatotoxicity of MTX in psoriasis.

In conclusion, although factors including study design, missed diagnosis, and the small number of specific genotype carriers may limit the generalizability of the study, results from this study add useful information to the evidence regarding the efficacy and hepatotoxicity of the low-dose MTX in patients with psoriasis with and without arthritis. Also, this is the first study to assess the potential association between *MTHFR* polymorphism and the susceptibility to PsA in the Han Chinese population. These observations contribute to our growing knowledge base of pharmacogenetics of psoriasis and PsA and personalized therapies.

## Data Availability Statement

The data presented in the study are deposited in the European Variation Archive database, accession number: PRJEB51117.

## Ethics Statement

The studies involving human participants were reviewed and approved by the Medical Ethics Committee of Huashan Hospital, Fudan University. The patients/participants provided their written informed consent to participate in this study.

## Author Contributions

JZ, ZW, XF, ZZhe, ZZha, and KYan designed the study. JZ and LT collected blood samples. JZ, NY, and KYan wrote the manuscript. JZ, ZW, ZZha, LT, LH, QH, XF, KYang, and GH contributed to the acquisition, analysis, or interpretation of data. ZW, ZZha, ZZhe, and KYan obtained the funding. JZ and ZZha contributed to the revision of the manuscript. KYan and ZZha are available post-publication to respond to any queries or critiques. All authors read and approved the final manuscript.

## Funding

This work was supported by the National Natural Science Foundation of China (Grant Nos. 81773322, 81673080, 81673073, 81974471, and 82173420), the Key Project in Basic Research Advocated by Shanghai Science and Technology Commission (Grant No. 13JC1402300), and the Clinical Research Plan of SHDC (Grant Nos. SHDC2020CR6022 and SHDC2020CR1014B). Funding sources had no involvement in the study design, the collection, analysis, or interpretation of the data, the writing of the report, and the decision to submit for publication.

## Conflict of Interest

The authors declare that the research was conducted in the absence of any commercial or financial relationships that could be construed as a potential conflict of interest.

## Publisher's Note

All claims expressed in this article are solely those of the authors and do not necessarily represent those of their affiliated organizations, or those of the publisher, the editors and the reviewers. Any product that may be evaluated in this article, or claim that may be made by its manufacturer, is not guaranteed or endorsed by the publisher.

## Abbreviations

ALT: alanine aminotransferase
AST: aspartate aminotransferase
BMI: body mass index
BSA: body surface area
DBIL: direct bilirubin
Hcy: homocysteine
HLA: human leukocyte antigen
MTHFR: methylenetetrahydrofolate reductase
MTX: methotrexate
PsA: psoriatic arthritis
PsO: psoriasis without psoriatic arthritis
PASI: Psoriasis Area and Severity Index
RA: rheumatic arthritis
SNP: single nucleotide polymorphism
TBIL: total bilirubin
TNF-α: tumor necrosis factor-α.
