# Supplementary material for: MTHFR Gene Polymorphism Association With Psoriatic Arthritis Risk and the Efficacy and Hepatotoxicity of Methotrexate in Psoriasis
Source: Front Med (Lausanne). 2022 Apr 11;9:869912. doi: 10.3389/fmed.2022.869912 (PMC9035632; doi:10.3389/fmed.2022.869912)
Supplement: Supplementary file 1 [file Table_1.DOCX]

Supplementary Material

**Supplementary Table S1. Supplementary clinical characteristics of 163 patients with PsA**

| **No.** | **Swollen or tender joints** | | | **Psoriasis^a^** | **Nail involvement^b^** | **RF negative** | **Dactylitis** |
| --- | --- | --- | --- | --- | --- | --- | --- |
|  | **Peripheral** | **Enthesitis** | **Axial** |  |  |  |  |
| Sum (n=163) | 132 (81.0%) | 46 (28.2%) | 24 (14.7%) | 163 (100.0%) | 152 (93.3%) | 159 (97.5%) | 66 (40.5%) |
| 1 | No | No | No | Yes | Yes | (-) | Yes |
| 2 | Yes | Yes | No | Yes | Yes | (-) | No |
| 3 | Yes | No | No | Yes | Yes | (-) | No |
| 4 | Yes | No | No | Yes | Yes | (-) | No |
| 5 | No | Yes | No | Yes | Yes | (-) | Yes |
| 6 | Yes | No | No | Yes | Yes | (-) | No |
| 7 | Yes | No | No | Yes | Yes | (-) | No |
| 8 | Yes | No | No | Yes | Yes | (-) | No |
| 9 | Yes | Yes | No | Yes | Yes | (-) | Yes |
| 10 | Yes | No | No | Yes | Yes | (-) | No |
| 11 | No | Yes | No | Yes | Yes | (-) | Yes |
| 12 | Yes | No | Yes | Yes | Yes | (-) | No |
| 13 | Yes | No | No | Yes | Yes | (-) | No |
| 14 | Yes | No | No | Yes | Yes | (+) | No |
| 15 | No | No | No | Yes | Yes | (-) | Yes |
| 16 | Yes | No | No | Yes | Yes | (-) | No |
| 17 | No | No | No | Yes | Yes | (-) | Yes |
| 18 | No | No | No | Yes | No | (-) | Yes |
| 19 | Yes | Yes | No | Yes | Yes | (-) | No |
| 20 | Yes | No | No | Yes | Yes | (-) | No |
| 21 | Yes | No | No | Yes | No | (-) | No |
| 22 | Yes | No | No | Yes | Yes | (-) | No |
| 23 | No | No | No | Yes | Yes | (-) | No |
| 24 | No | No | No | Yes | Yes | (-) | Yes |
| 25 | Yes | Yes | No | Yes | Yes | (-) | Yes |
| 26 | Yes | Yes | No | Yes | Yes | (-) | Yes |
| 27 | Yes | No | No | Yes | Yes | (-) | No |
| 28 | No | No | Yes | Yes | Yes | (-) | Yes |
| 29 | No | Yes | Yes | Yes | Yes | (-) | Yes |
| 30 | Yes | No | No | Yes | Yes | (+) | No |
| 31 | Yes | No | No | Yes | Yes | (-) | No |
| 32 | Yes | No | No | Yes | Yes | (-) | No |
| 33 | Yes | No | No | Yes | Yes | (-) | No |
| 34 | Yes | No | Yes | Yes | Yes | (-) | No |
| 35 | Yes | Yes | No | Yes | Yes | (-) | Yes |
| 36 | Yes | No | No | Yes | Yes | (-) | Yes |
| 37 | Yes | Yes | No | Yes | Yes | (-) | Yes |
| 38 | Yes | No | No | Yes | No | (-) | No |
| 39 | Yes | No | No | Yes | Yes | (-) | No |
| 40 | Yes | No | No | Yes | Yes | (-) | No |
| 41 | Yes | No | No | Yes | Yes | (-) | No |
| 42 | Yes | No | No | Yes | Yes | (-) | No |
| 43 | Yes | Yes | No | Yes | Yes | (-) | Yes |
| 44 | Yes | No | Yes | Yes | Yes | (-) | Yes |
| 45 | Yes | No | No | Yes | Yes | (-) | No |
| 46 | Yes | Yes | No | Yes | Yes | (-) | Yes |
| 47 | Yes | No | No | Yes | Yes | (-) | No |
| 48 | Yes | No | No | Yes | Yes | (-) | No |
| 49 | Yes | No | No | Yes | Yes | (-) | No |
| 50 | Yes | Yes | No | Yes | Yes | (-) | Yes |
| 51 | Yes | No | No | Yes | Yes | (-) | No |
| 52 | Yes | Yes | No | Yes | Yes | (-) | Yes |
| 53 | Yes | Yes | Yes | Yes | Yes | (-) | Yes |
| 54 | Yes | No | No | Yes | Yes | (-) | No |
| 55 | Yes | No | No | Yes | Yes | (-) | No |
| 56 | No | No | No | Yes | Yes | (-) | Yes |
| 57 | Yes | No | No | Yes | Yes | (-) | No |
| 58 | No | No | No | Yes | Yes | (-) | Yes |
| 59 | Yes | No | No | Yes | Yes | (-) | No |
| 60 | Yes | No | No | Yes | Yes | (-) | No |
| 61 | Yes | Yes | Yes | Yes | Yes | (-) | Yes |
| 62 | Yes | No | No | Yes | Yes | (-) | No |
| 63 | Yes | No | No | Yes | Yes | (-) | No |
| 64 | Yes | No | No | Yes | Yes | (-) | No |
| 65 | Yes | Yes | Yes | Yes | No | (-) | Yes |
| 66 | Yes | No | No | Yes | Yes | (-) | Yes |
| 67 | Yes | No | No | Yes | Yes | (-) | No |
| 68 | Yes | Yes | No | Yes | Yes | (-) | Yes |
| 69 | Yes | No | No | Yes | Yes | (-) | No |
| 70 | Yes | Yes | No | Yes | Yes | (-) | No |
| 71 | Yes | No | No | Yes | Yes | (-) | Yes |
| 72 | Yes | No | No | Yes | Yes | (-) | No |
| 73 | No | No | No | Yes | Yes | (-) | Yes |
| 74 | Yes | No | No | Yes | Yes | (-) | No |
| 75 | Yes | Yes | No | Yes | Yes | (-) | No |
| 76 | Yes | No | No | Yes | Yes | (-) | No |
| 77 | Yes | Yes | No | Yes | Yes | (-) | Yes |
| 78 | Yes | No | No | Yes | Yes | (-) | Yes |
| 79 | Yes | Yes | No | Yes | Yes | (-) | No |
| 80 | Yes | No | No | Yes | Yes | (-) | No |
| 81 | Yes | No | No | Yes | Yes | (-) | No |
| 82 | Yes | No | No | Yes | No | (-) | Yes |
| 83 | Yes | No | No | Yes | Yes | (-) | Yes |
| 84 | No | No | No | Yes | Yes | (-) | Yes |
| 85 | Yes | No | No | Yes | Yes | (-) | No |
| 86 | No | No | No | Yes | Yes | (-) | Yes |
| 87 | Yes | Yes | No | Yes | Yes | (-) | Yes |
| 88 | Yes | Yes | No | Yes | Yes | (-) | No |
| 89 | Yes | No | No | Yes | Yes | (-) | Yes |
| 90 | Yes | Yes | No | Yes | Yes | (-) | Yes |
| 91 | Yes | Yes | No | Yes | Yes | (-) | Yes |
| 92 | Yes | Yes | Yes | Yes | Yes | (-) | Yes |
| 93 | Yes | No | No | Yes | Yes | (-) | No |
| 94 | Yes | No | No | Yes | Yes | (-) | No |
| 95 | No | No | No | Yes | Yes | (-) | Yes |
| 96 | Yes | No | No | Yes | Yes | (-) | Yes |
| 97 | Yes | No | No | Yes | Yes | (-) | No |
| 98 | No | No | No | Yes | Yes | (-) | Yes |
| 99 | Yes | No | No | Yes | Yes | (-) | No |
| 100 | Yes | No | No | Yes | Yes | (-) | No |
| 101 | Yes | No | No | Yes | Yes | (-) | No |
| 102 | Yes | Yes | No | Yes | Yes | (-) | Yes |
| 103 | Yes | Yes | No | Yes | Yes | (-) | No |
| 104 | Yes | No | No | Yes | Yes | (-) | No |
| 105 | Yes | Yes | No | Yes | Yes | (-) | No |
| 106 | No | No | No | Yes | Yes | (-) | Yes |
| 107 | Yes | Yes | No | Yes | Yes | (+) | Yes |
| 108 | Yes | Yes | No | Yes | Yes | (-) | No |
| 109 | Yes | No | No | Yes | Yes | (-) | No |
| 110 | Yes | Yes | No | Yes | No | (-) | Yes |
| 111 | Yes | No | No | Yes | Yes | (-) | No |
| 112 | Yes | No | No | Yes | Yes | (-) | No |
| 113 | Yes | Yes | No | Yes | Yes | (-) | No |
| 114 | Yes | No | No | Yes | Yes | (-) | No |
| 115 | Yes | No | Yes | Yes | Yes | (-) | No |
| 116 | No | No | No | Yes | Yes | (-) | Yes |
| 117 | No | Yes | Yes | Yes | Yes | (-) | Yes |
| 118 | Yes | No | No | Yes | Yes | (-) | No |
| 119 | Yes | No | No | Yes | Yes | (-) | No |
| 120 | Yes | No | No | Yes | Yes | (-) | No |
| 121 | Yes | Yes | No | Yes | Yes | (-) | No |
| 122 | Yes | No | Yes | Yes | Yes | (-) | No |
| 123 | No | Yes | No | Yes | Yes | (-) | Yes |
| 124 | No | Yes | Yes | Yes | Yes | (-) | Yes |
| 125 | Yes | No | No | Yes | Yes | (-) | No |
| 126 | Yes | No | No | Yes | Yes | (-) | No |
| 127 | Yes | Yes | No | Yes | Yes | (-) | Yes |
| 128 | Yes | No | Yes | Yes | Yes | (-) | No |
| 129 | Yes | No | No | Yes | Yes | (-) | No |
| 130 | No | Yes | Yes | Yes | Yes | (-) | Yes |
| 131 | Yes | Yes | No | Yes | Yes | (-) | No |
| 132 | No | No | Yes | Yes | Yes | (-) | Yes |
| 133 | Yes | Yes | Yes | Yes | Yes | (+) | Yes |
| 134 | No | No | No | Yes | No | (-) | No |
| 135 | No | Yes | No | Yes | Yes | (-) | Yes |
| 136 | Yes | No | Yes | Yes | Yes | (-) | No |
| 137 | Yes | No | Yes | Yes | Yes | (-) | No |
| 138 | Yes | No | No | Yes | Yes | (-) | No |
| 139 | Yes | No | No | Yes | Yes | (-) | No |
| 140 | Yes | No | No | Yes | Yes | (-) | No |
| 141 | Yes | No | Yes | Yes | Yes | (-) | Yes |
| 142 | No | No | No | Yes | Yes | (-) | No |
| 143 | Yes | No | No | Yes | Yes | (-) | No |
| 144 | Yes | No | No | Yes | Yes | (-) | Yes |
| 145 | Yes | Yes | Yes | Yes | Yes | (-) | No |
| 146 | No | No | No | Yes | No | (-) | No |
| 147 | No | No | No | Yes | Yes | (-) | Yes |
| 148 | Yes | No | Yes | Yes | Yes | (-) | No |
| 149 | Yes | Yes | Yes | Yes | Yes | (-) | Yes |
| 150 | Yes | No | No | Yes | Yes | (-) | Yes |
| 151 | No | No | No | Yes | Yes | (-) | No |
| 152 | Yes | No | No | Yes | No | (-) | No |
| 153 | Yes | Yes | No | Yes | Yes | (-) | No |
| 154 | Yes | No | No | Yes | Yes | (-) | No |
| 155 | Yes | No | No | Yes | Yes | (-) | No |
| 156 | Yes | No | No | Yes | Yes | (-) | No |
| 157 | Yes | No | No | Yes | No | (-) | Yes |
| 158 | Yes | No | Yes | Yes | No | (-) | Yes |
| 159 | Yes | No | No | Yes | Yes | (-) | No |
| 160 | No | No | No | Yes | Yes | (-) | Yes |
| 161 | Yes | No | No | Yes | Yes | (-) | Yes |
| 162 | Yes | No | No | Yes | Yes | (-) | Yes |
| 163 | Yes | No | No | Yes | Yes | (-) | No |

^a^ Presence of psoriasis or its familial history.

^b^ Typical psoriatic nail dystrophy (e.g., onycholysis, pitting, or hyperkeratosis) according to observation during monthly physical examination.

**Supplementary Table S2. The distribution of *MTHFR* SNP rs1801133 and rs1801131 in 309 patients of psoriasis**

|  | **Genotype** | **N (frequency)** | **Allele** | **N (frequency)** | **HWE** |
| --- | --- | --- | --- | --- | --- |
| **rs1801133** | CC (Wild type) | 111 (35.9%) | C | 367 (59.4%) |  |
|  | CT | 145 (46.9%) |  |  |  |
|  | TT | 53 (17.2%) | T | 251 (40.6%) | 0.6324 |
| **rs1801131** | CC | 8 (2.6%) | C | 104 (16.8%) |  |
|  | CT | 88 (28.5%) |  |  |  |
|  | TT (Wild type) | 213 (68.9%) | T | 514 (83.2%) | 0.7602 |
| **Healthy Controls** | CC | 341 (33.1) | C | 1174 (56.9%) |  |
| **(rs1801133)** | CT | 492 (47.7) |  |  |  |
|  | TT | 198 (19.2) | T | 888 (43.1%) | 0.3883 |
